# Time patterns in online survey completion and offline psychological symptoms among college students in China

**DOI:** 10.3389/fpubh.2024.1430256

**Published:** 2024-07-23

**Authors:** Yiyang Liu, Shuang Xu, Peiyue Yang, Haolou Feng, Shaoshuai Wu, Xiaoping Yin, Guowei Zhang, Qi Lu, Zhichen Dong, Shunfei Li, Hongguang Chen

**Affiliations:** ^1^NHC Key Laboratory of Mental Health, National Clinical Research Center for Mental Disorders, Peking University Sixth Hospital, Institute of Mental Health, Peking University, Beijing, China; ^2^Hebei College of Science and Technology, Baoding, China; ^3^Hebei Key Laboratory of Precise Imaging of Inflammation Related Tumors, Affiliated Hospital of Hebei University, Baoding, China; ^4^College of Chinese Medicine, Hebei University, Baoding, China; ^5^Health Science Center, Peking University, Beijing, China; ^6^Chinese PLA General Hospital, Beijing, China

**Keywords:** online behavior, psychological symptoms, digital public health, college students, epidemiology

## Abstract

**Background:**

Online psychological surveys allow for swift data collection among college students, thus providing a foundation for psychological interventions, particularly during emergent public health events. However, the association between online survey completion behaviors and offline psychological symptoms has yet to be explored.

**Methods:**

A large-scale web-based survey was conducted from December 31, 2022, to January 7, 2023, involving 22,624 participants. Psychological symptoms were assessed using standardized measures, while the time taken to complete the survey and the time of completion were recorded by the online survey platform.

**Results:**

As the time duration increased, the prevalence of anxiety, depression, insomnia, and PTSD also increased significantly (*P*_for trend_ < 0.001). The highest odds ratios were observed in the longer duration group. Only a longer duration was significantly associated with PTSD. The time period for completing the questionnaire from 7 p.m. to 10 p.m. was found to be significantly linked with anxiety symptoms and depression symptoms. Conversely, completing the questionnaire at other times was specifically associated with anxiety symptoms and insomnia symptoms. The prolonged duration needed to complete the questionnaire was more closely related to the comorbidity of anxiety, depression, and insomnia than to the comorbidity of those symptoms with PTSD. When questionnaires were completed during other times, specifically referring to the late-night and early morning hours, individuals were more likely to exhibit comorbid symptoms of insomnia.

**Conclusion:**

The study identified the specific associations between time durations, time points for completing online survey, and psychological symptoms/comorbidity among college students. Further exploration of their causal relationships and the underlying mechanisms is warranted.

## Background

Facing the challenges of transitioning from adolescence to adulthood, college students encounter various psychological issues that significantly impact their well-being. This is particularly pronounced amidst a large-scale outbreak such as the COVID-19 pandemic (1). These challenges are multifaceted, influenced by academic pressures, social isolation, financial concerns, uncertainty about the future, and the overall pandemic impact on daily life (2, 3). Understanding the psychological state of college students in this context is crucial for effective interventions and support, as well as for devising psychological intervention and prevention measures. As the primary demographic of internet users, college students demonstrate a notable willingness and capability to participate in online surveys, surpassing other age groups. This presents a unique opportunity to explore their psychological well-being in depth through online surveys (4, 5). Online surveys offer distinct advantages in convenience, timeliness, and cost-effectiveness, facilitating swift data collection and laying the groundwork for psychological interventions (3, 6). Beyond capturing psychological symptoms, surveys can also record respondent behavior, such as time points and time duration to finish a survey. Time duration and time point, as parallel data in electronically based research, are highly objective data and information that are extremely easy to be overlooked by researchers and participants alike. However, these data may carry crucial content related to the early identification of mental illnesses (7, 8). Previous research on behaviors related to online survey completion duration has primarily focused on questionnaire quality control, with very few reports on the relationship between time points and psychological conditions (9, 10). Given this backdrop, this study employs a large-scale web-based survey to investigate the association between online survey completion behaviors and offline psychological symptoms among college students.

## Materials and methods

### Study design

This cross-sectional study was conducted in China from December 31, 2022, to January 7, 2023. The online questionnaire, accessed through a web-based platform with a Quick Response code (QR code), was sent to student counselors at colleges located in Hebei Province. The student counselors then forwarded the QR code to the WeChat group of college students in each department, allowing them to voluntarily complete the questionnaire. Regarding the questionnaire completion deadline: it was recommended to complete the questionnaire within 2 days of receiving the QR code. There were no specific requirements for the questionnaire responding time, nor were there prompts to complete it as soon as possible.

### Ethical considerations

A total of 25,737 survey responses were collected and analyzed after obtaining electronic informed written consent from all respondents. All data were deidentified and anonymized. The Ethics Committee of Peking University Sixth Hospital approved the study (Approval number: 2022-9-5-1).

### Measurements

#### Demographic characteristics and COVID-19 infection

A self-designed questionnaire was used to obtain sociodemographic data, including gender, age, education, discipline classification, and information on COVID-19 infection, categorized as not infected, recovered from infection, and unrecovered from infection.

#### Time duration and point to finish the survey

The online platform for this study automatically recorded the specific time point and time spent by respondents to complete the online survey.

Time duration to finish the survey was defined as the time duration from scanning the survey QR code and entering to start filling out the questionnaire, to the completion of the questionnaire. The time duration was divided into four intervals using quartiles after excluding those with a response time less than the 10th percentile and greater than the 90th percentile: shorter (<=198 s), short (> 198 s & < =247 s), long (>247 s & < =310 s), and longer (>310 s). Due to the limited research on the relationship between the time taken to complete online questionnaire and psychological symptoms, there are currently no grouping methods available for reference. Therefore, to consider the sample balance between the groups, this study adopted the quartile grouping method. Utilizing quartiles for time duration classification can offer several benefits, such as providing a balanced distribution of data points and enabling the comparison of different time intervals within the dataset. By categorizing time duration into quartiles, we can identify patterns, trends, and variations among different segments of the data, facilitating a more nuanced understanding of the relationship between time duration and psychological symptoms.

Time point to finish the survey was defined as the time at which the respondent completed the survey. This study categorized the participants’ response time points into four subgroups: from 11 a.m. to 2 p.m. representing the timely completion group, from 3 p.m. to 6 p.m. representing the short delay group, from 7 p.m. to 10 p.m. representing the long delay group, and other times from 11 p.m. to the morning before 10 a.m. of the next day of the survey distribution, representing the longer delay group. The survey questionnaire was distributed to college students on the same day, primarily between 11 a.m. and 2 p.m. Therefore, based on the distribution time of the questionnaire, response time, and participants’ daily schedules, the time points were classified into the aforementioned four categories.

### Psychological symptoms

#### Generalized anxiety disorder 7-item scale

This scale consisted of seven items used to screen for anxiety symptoms and evaluate their severity (11, 12). There were four degrees for each item (0-not at all, 1-some of the time; 2-more than half the time; 3-nearly every day). The GAD-7 score ranges from 0 to 21, with a total score of 0–4 rated as no anxiety, 5–9 rated as mild, 10–14 rated as moderate, and 15 or more rated as severe anxiety. The Cronbach’s alpha coefficient of the scale in this study was 0.926.

#### Patient health questionnaire-9

This scale consisted of nine items used to screen for depressive symptoms and evaluate their severity (13, 14). There were four degrees for each item (0-not at all, 1-some of the time; 2-more than half the time; 3-nearly every day). The PHQ-9 score ranges from 0 to 27, with a total score of 0–4 rated as no depression, 5–9 rated as mild, 10–14 rated as moderate, 15–19 rated as moderate to severe, and 20 or more rated as severe depression. The Cronbach’s alpha coefficient of the scale in this study was 0.895.

#### Insomnia severity index

The ISI scale consisted of seven items, each with 0–4 points, and was used to screen for insomnia symptoms and evaluate their severity (15, 16). The total score ranges from 0 to 28, with a score of 0–7 rated as no insomnia, 8–14 rated as mild, 15–21 rated as moderate, and 22 or more rated as severe insomnia. The Cronbach’s alpha coefficient of the scale in this study was 0.905.

#### Impact of events scale-revised

The IES-R is a 22-item self-report instrument that corresponds to DSM-IV symptoms of PTSD (17, 18). For every item, individuals indicate the level of distress they experienced during the past 7 days, using a scale from 0 (not at all) to 4 (extremely). A score of 0–23 is rated as usual psychological impact, 24–32 as mild, 33–36 as moderate, and 37 or more as severe psychological impact. The Cronbach’s alpha coefficient of the scale in this study was 0.953.

### Psychological comorbidity

Psychological comorbidity was defined as individuals simultaneously meeting criteria for two or more psychological symptoms according to the cutoff points of the respective scales mentioned above.

### Statistical analyses

Descriptive analysis was performed using absolute and prevalence for categorical variables. The chi-square test was utilized to compare proportions, while multivariable logistic regression models were applied to calculate the odds ratio (OR) and 95% confidence interval (CI) of time point and duration in relation to psychological symptoms among college students. These analyses were adjusted for variables such as age, gender, education level, major, daily online activity, personal COVID-19 infection, and family COVID-19 infection. By adjusting for these factors, the study aimed to provide a more accurate understanding of the relationship between temporal factors and psychological symptoms in the college student population. Furthermore, the study employed multinomial logistic regression analysis to delve deeper into the intricate relationship between time points, time durations, and a spectrum of psychological symptoms, encompassing anxiety, depression, insomnia, and PTSD, both as standalone conditions and in comorbidity with each other. Multinomial logistic regression, a sophisticated classification technique, extends the logistic regression algorithm to address multiclass possible outcome problems. It enables the prediction of probabilities associated with categorically dependent variables that encompass two or more possible outcome classes. This approach allowed for a nuanced understanding of how different time points and durations correlate with specific psychological symptoms and their comorbidities among college students. All statistical tests employed were two-tailed with a significance level set at *p* < 0.05, ensuring robustness and reliability in the analysis. The data underwent thorough examination using STATA 14.0 (StataCorp, College Station, Texas, United States).

## Results

### Sociodemographic characteristics

A total of 20,085 college students with a median age of 20.4 years (IQR, 19.3–21.5) were included in the final analysis after excluding 5,652 cases with incomplete information or with a response time less than the 10th percentile and greater than the 90th percentile. The self-reported prevalence of anxiety symptoms, depression symptoms, insomnia symptoms, and PTSD symptoms were 12.0, 24.8, 11.0, and 7.4%, respectively. The respondents had an average age of 20.48 ± 1.48 years, with females comprising 52.62% of the sample. Medical students accounted for 11.57% of the participants, while undergraduates made up 70.64%. The highest proportion of respondents, 40.02%, reported spending 3–5 h online daily, followed by 27.78% spending less than 3 h. Regarding COVID-19 infection status, 64.97% reported recovery after infection, while 19.78% had not been infected. In terms of family COVID-19 infections, the highest proportion, 37.68%, reported that their entire family had been infected.

### Time duration/time point to finish the survey and psychological symptoms

The median time taken by all subjects to complete the questionnaire was 247 s (IQR, 198–310). Females took longer to complete the questionnaire (median, 260, IQR, 209–321) than males (median, 232, IQR, 189–293, *p* < 0.001). Regarding time duration, as the time duration increased, the prevalence of anxiety, depression, insomnia, and PTSD also increased significantly (*p*_for trend_ < 0.001, see Table 1). Specifically, anxiety, depression, insomnia, and PTSD were most prevalent among respondents in the longer duration category. Participants who completed the survey from 7 p.m. to 10 p.m. exhibited significantly higher prevalence rates of anxiety and depression compared to those who completed it from 11 a.m. to 2 p.m. and from 3 p.m. to 6 p.m. However, there was no significant difference in the prevalence of insomnia or PTSD between these time points.

**Table 1:** Correlation between time duration/time point to finish the survey and psychological symptoms among 22,624 college students.

	Total *N*	Anxiety *n* (%)	Depression *n* (%)	Insomnia *n* (%)	PTSD *n* (%)
Time duration					
Shorter	5,035	472 (9.37)	935 (18.57)	394 (7.83)	331 (6.57)
Short	5,112	534 (10.45)	1,170 (22.89)	549 (10.74)	360 (7.04)
Long	4,978	639 (12.84)	1,328 (26.68)	590 (11.85)	371 (7.45)
Longer	1,960	761 (15.34)	1,539 (31.03)	683 (13.77)	421 (8.49)
*p*_for trend_		<0.001	<0.001	<0.001	<0.001
Time point					
11 a.m. to 2 p.m.	15,773	1906 (12.08)	3,967 (25.15)	1733 (10.99)	1,183 (7.5)
3 p.m. to 6 p.m.	2,690	282 (10.48)	593 (22.04)	280 (10.41)	190 (7.06)
7 p.m. to 10 p.m.	942	125 (13.27)	240 (25.48)	109 (11.57)	66 (7.01)
others	680	93 (13.68)	172 (25.29)	94 (13.82)	44 (6.47)
*p*		0.027	0.006	0.079	0.628

### Associations between time duration/time point to finish the survey and each psychological symptom examined by multivariable logistic regression

The data indicated that time durations were associated with a significantly higher risk for anxiety symptoms, depression symptoms, and insomnia symptoms. The highest ORs were observed in the longer duration group, with an adjusted OR of 1.85 (95% CI: 1.63–2.09, see Table 2) in the anxiety group, 2.09 (95% CI: 1.89–2.30) in the depression group, and 1.99 (95% CI: 1.74–2.28) in the insomnia group.

**Table 2:** Associations between time duration/time point and each psychological symptom examined by multivariable logistic regression.

	Anxiety Adjusted OR (95%CI)^a^	*p*	Depression Adjusted OR (95%CI)^a^	*p*	Insomnia Adjusted OR (95%CI)^a^	*p*	PTSD Adjusted OR (95%CI)^a^	*p*
Time duration to finish								
Short	1.15 (1.01–1.31)	0.038	1.33 (1.21–1.47)	<0.001	1.46 (1.27–1.68)	<0.001	1.09 (0.93–1.27)	0.289
Long	1.48 (1.30–1.69)	<0.001	1.66 (1.51–1.83)	<0.001	1.67 (1.46–1.92)	<0.001	1.15 (0.99–1.35)	0.073
Longer	1.85 (1.63–2.09)	<0.001	2.09 (1.89–2.30)	<0.001	1.99 (1.74–2.28)	<0.001	1.33 (1.14–1.55)	<0.001
Time point to finish								
3 p.m. to 6 p.m.	0.93 (0.82–1.07)	0.323	0.92 (0.83–1.02)	0.124	1.01 (0.88–1.16)	0.882	1.02 (0.87–1.20)	0.823
7 p.m. to 10 p.m.	1.31 (1.07–1.60)	0.008	1.21 (1.03–1.42)	0.017	1.11 (0.90–1.38)	0.318	1.06 (0.81–1.38)	0.663
Others	1.30 (1.03–1.63)	0.024	1.12 (0.94–1.35)	0.214	1.44 (1.15–1.81)	0.002	0.96 (0.70–1.32)	0.805

a Adjusted for age, gender, education level, major, online time every day, personal COVID-19 infection and family COVID-19 infection in each model.

Moreover, the time point to finish the questionnaire from 7 p.m. to 10 p.m. was significantly associated with anxiety symptoms (adjusted OR: 1.31, 95% CI: 1.07–1.60) and depression symptoms (adjusted OR: 1.21, 95% CI: 1.03–1.42). Conversely, completing the questionnaire at other times was specifically associated with anxiety symptoms (adjusted OR: 1.30, 95% CI: 1.03–1.63) and insomnia symptoms (adjusted OR: 1.44, 95% CI: 1.15–1.81). Only a longer duration was significantly associated with PTSD, with an adjusted OR of 1.33 (95% CI: 1.14–1.55). However, no statistically significant associations were found for short and long time durations. Additionally, the time point to finish the questionnaire showed no statistically significant association with PTSD.

### Associations between time duration/time point to finish the survey and psychological comorbidity

Table 3 illustrates the distribution of psychological comorbidities across different time durations, revealing distinct patterns in symptom combinations. In the anxiety category, as time duration increases, the proportion of individuals with anxiety only and comorbidity with other symptoms generally increases, except for those with comorbidity with PTSD. For depression, with prolonged time duration, there is a noticeable increase in the proportion of individuals with depression only and comorbidity with other symptoms. In the insomnia category, as time duration extends, there is an increase observed only in the proportion of individuals experiencing insomnia only and comorbidity with depression. In the PTSD category, as time duration increases, there is a slight increase noted only in the proportion of those with PTSD only. No significant differences were observed between psychological comorbidity and the time point to finish the questionnaire (data not shown).

**Table 3:** Correlations between time duration to finish the survey and psychological comorbidity among 6,723 college students with psychological symptom.

	Time duration				*p*
	Shorter *n* (%)	Short *n* (%)	Long *n* (%)	Longer *n* (%)	
Anxiety					
Anxiety only	19 (13.48)	29 (20.57)	37 (26.24)	56 (39.72)	<0.001
Comorbidity depression	132 (16.84)	158 (20.15)	221 (28.19)	273 (34.82)	
Comorbidity insomnia	78 (15.66)	107 (21.49)	142 (28.51)	171 (34.34)	
Comorbidity PTSD	243 (24.72)	240 (24.42)	239 (24.31)	261 (26.55)	
Depression					
Depression only	406 (19.95)	503 (24.72)	542 (26.63)	584 (28.7)	<0.001
Comorbidity anxiety	132 (16.84)	158 (20.15)	221 (28.19)	273 (34.82)	
Comorbidity insomnia	124 (13.03)	222 (23.32)	266 (27.94)	340 (35.71)	
Comorbidity PTSD	273 (22.73)	287 (23.9)	299 (24.9)	342 (28.48)	
Insomnia					
Insomnia only	44 (14.57)	76 (25.17)	86 (28.48)	96 (31.79)	<0.001
Comorbidity anxiety	4 (19.05)	7 (33.33)	8 (38.1)	2 (9.52)	
Comorbidity depression	124 (13.03)	222 (23.32)	266 (27.94)	340 (35.71)	
Comorbidity PTSD	222 (23.59)	244 (25.93)	230 (24.44)	245 (26.04)	
PTSD					
PTSD only	36 (18.85)	53 (27.75)	49 (25.65)	53 (27.75)	0.014
Comorbidity anxiety	4 (30.77)	2 (15.38)	1 (7.69)	6 (46.15)	
Comorbidity depression	69 (20.41)	61 (18.05)	91 (26.92)	117 (34.62)	
Comorbidity insomnia	222 (23.59)	244 (25.93)	230 (24.44)	245 (26.04)	

### Associations between time duration/time point to finish the survey and psychological comorbidity examined by multinomial logistic regression

**Figure 1:**
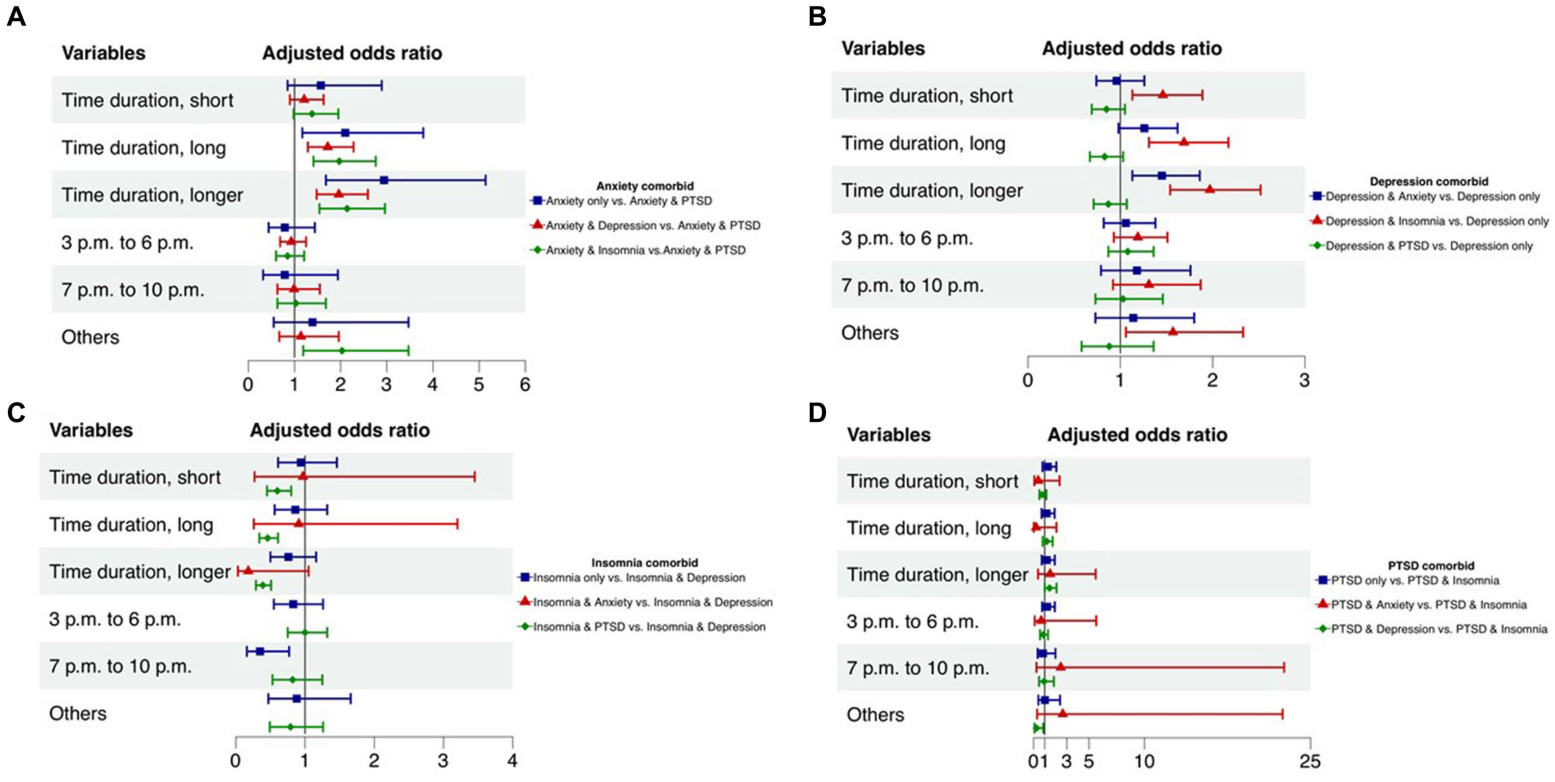
Correlations between time duration and psychological comorbidity examined by multinomial logistic regression*. “*” The odds ratio was adjusted for age, gender, education level, major, online time every day, personal COVID-19 infection and family COVID-19 infection in each model. **(A)** Correlations between time duration and anxiety comorbidity. **(B)** Correlations between time duration and depression comorbidity. **(C)** Correlations between time duration and insomnia comorbidity. **(D)** Correlations between time duration and PTSD comorbidity.

Figure 1 presents findings on the relationship between time durations or points and psychological comorbidities. For individuals with anxiety, prolonged time durations significantly increase the likelihood of experiencing anxiety alone, comorbidty with depression, and insomnia. Notably, there were no notable disparities in odds ratios observed across different time points, except within the “Others” category. For individuals with depression, prolonged time durations are significantly associated with a higher risk of comorbidity with anxiety and insomnia. The association between time points and depression comorbidities varies, with depression and insomnia showing significance during time points categorized as “Others.” Regarding individuals with insomnia, prolonged time durations are associated solely with a higher risk of comorbidity with depression compared to PTSD. Respondents participating from 7 p.m. to 10 p.m. exhibit a significantly higher risk of comorbidity with depression. For individuals with PTSD, longer time durations significantly increase the risk of comorbidity with depression. Remarkably, there is a significant increase in the risk of comorbidity with insomnia within the “Others” category.

## Discussion

### Online survey completion behaviors and offline psychological symptoms

The findings highlight a significant correlation: longer time duration to finish the survey are associated with an increased risk of psychological symptoms, which may be attributed to the cognitive impairments caused by these symptoms. Previous studies have reported that psychological symptoms can impede various cognitive functions. Anxiety, for instance, has been linked to impairments in attentional control and working memory (19, 20), with high levels negatively affecting attentional performance and cognitive flexibility (21). Similarly, depression is known to cause cognitive deficits, such as difficulties in sustained attention, information processing, and episodic memory (22), along with impaired executive functions like inhibition and set-shifting (23). Insomnia has also been shown to be connected to impairments in attention, concentration, working memory, and overall cognitive performance (24, 25), with sleep deprivation exacerbating these issues by impairing executive functions, including decision-making and response inhibition (23). PTSD presents with deficits in attention, working memory, and executive functions due to impaired cognitive control (26, 27), where symptoms like hypervigilance can disrupt selective attention and cognitive task performance. Furthermore, the study also found an association between the timing of completing the survey and psychological symptoms. Especially noteworthy was the strong correlation between participants who completed the questionnaire between 7 pm and 10 pm and symptoms of anxiety and depression. Specifically, there could be two main reasons. First, individuals with psychological symptoms may have difficulties in emotional regulation and concentrating attention, but they often engage in negative thinking patterns, thereby experiencing reduced self-efficacy. To a large extent, this could make it difficult for individuals to focus or to adopt a positive mindset to complete survey questionnaires promptly. Previous research has also reported that individuals with anxiety or depression tend to exhibit procrastination or delay behaviors (28–30). Additionally, anxiety and depression also exhibit a characteristic of being more severe during the day and lighter at night. This also partially explains the correlation between completion of the questionnaire at night and psychological symptoms.

### Online survey completion behaviors and offline psychological comorbidities

Additionally, the study also found that both time duration and time point to complete the survey are also correlated with the psychological comorbidities. Findings indicated that the duration was longer when anxiety, depression, and insomnia coexisted compared to when they coexisted with PTSD. This may be related to the following aspects: (1) Differences in neurobiological foundations: Symptoms of depression, anxiety, and insomnia may involve common neurobiological foundations, such as neurotransmitter imbalances and abnormal activity in neural circuits (22, 25, 31). These abnormalities are closely related to cognitive decline. On the other hand, PTSD is mainly associated with neurobiological changes triggered by traumatic experiences (32). (2) Mutual influence of symptoms: Symptoms of depression and anxiety can lead to difficulties in attention concentration, slowed thinking, and decision-making problems, resulting in cognitive decline. Insomnia can further exacerbate these cognitive impairments (24). In contrast, PTSD symptoms primarily involve the recall and reactions to traumatic events (33). While it can also lead to anxiety and insomnia, it typically does not directly impact immediate responsiveness and decision-making abilities as anxiety and depression might (34, 35). This does not imply that comorbid PTSD does not cause cognitive and physiological impairments; rather, it highlights that comorbidities like anxiety, depression, and insomnia may impose a different and more varied cognitive burden, collectively contributing to slower response and decision speeds (26, 36). Regarding the time point to complete the survey, when respondents reply during other times, specifically referring to the late-night and early morning hours, compared to their co-occurrence with other psychological symptoms, their co-occurrence with insomnia symptoms shows a stronger association. There might be several reasons for this phenomenon. Firstly, insomnia itself leads to decreased sleep quality, increasing individuals’ likelihood of wakefulness or difficulty falling asleep at night (37). Consequently, they may be awake during late night or early morning hours, allowing more time to complete the survey. Secondly, psychological disorders such as anxiety, depression, or PTSD disrupt daily routines, leading individuals to be more active at night or struggle with falling asleep in the evening (3, 38). Thirdly, co-occurring insomnia exacerbates these impairments in executive function, resulting in delayed completion (39, 40). Therefore, the combination of these factors may lead individuals with both anxiety, depression, or PTSD and insomnia to be more inclined to complete tasks during late night or early morning hours.

### Limitations

This study has several limitations. Firstly, convenience sampling was used for participant recruitment, potentially biasing the findings and limiting their applicability to broader populations. The cross-sectional design prevents establishing causal relationships between online questionnaire completion dynamics and offline psychological symptoms or comorbidity. Future research could employ longitudinal or experimental methods to uncover temporal relationships and infer causality, enhancing understanding of these phenomena. Additionally, the interval setting for “time point to finish the questionnaire” lacked relevant literature and was based on questionnaire distribution time ranges, necessitating caution in generalizing or comparing results. Furthermore, respondents’ response time and response points in the study may be influenced by various factors such as school work and school courses. To mitigate these influences, the questionnaires were uniformly distributed on Saturdays. However, it is still impossible to completely eliminate the impact of these factors. Despite these limitations, the study’s strengths include a large sample size, which enhances the persuasiveness and reliability of the results. This facilitates a more comprehensive understanding of the relationship between online survey completion dynamics and offline psychological symptoms/comorbidity, offering hypotheses for future research.

## Conclusion

This study addresses the specific associations between time durations, time points for completing online survey, and psychological symptoms/comorbidity among college students. Completion time in online surveys independently associates with respondents’ psychological symptoms, with longer durations indicating higher risk. Participants completing surveys from 7 pm to 10 pm showed higher risk of anxiety and depression. Additionally, the prolonged time to complete the survey correlated more strongly with the coexistence of anxiety, depression, and insomnia than with the comorbidity of these conditions with PTSD. These findings indicate that online survey completion behaviors could serve as potential indicators of psychological well-being among college students. While the study provides preliminary evidence for these associations, further research is needed to explore the causality and predictive value of these behaviors in relation to psychological symptoms and comorbidity.

## Data availability statement

The raw data supporting the conclusions of this article will be made available by the authors, without undue reservation.

## Ethics statement

The studies involving humans were approved by the Ethics Committee of Peking University Sixth Hospital. The studies were conducted in accordance with the local legislation and institutional requirements. The participants provided their written informed consent to participate in this study.

## Author contributions

YL: Formal analysis, Methodology, Visualization, Writing – original draft, Writing – review & editing. SX: Conceptualization, Supervision, Writing – original draft, Writing – review & editing. PY: Investigation, Project administration, Writing – original draft, Writing – review & editing. HF: Investigation, Project administration, Writing – original draft, Writing – review & editing. SW: Investigation, Project administration, Writing – original draft, Writing – review & editing. XY: Investigation, Project administration, Writing – original draft, Writing – review & editing. GZ: Investigation, Project administration, Writing – original draft, Writing – review & editing. QL: Writing – original draft, Writing – review & editing. ZD: Writing – original draft, Writing – review & editing. SL: Conceptualization, Formal analysis, Funding acquisition, Methodology, Visualization, Supervision, Writing – original draft, Writing – review & editing. HC: Conceptualization, Formal analysis, Funding acquisition, Methodology, Visualization, Supervision, Writing – original draft, Writing – review & editing.
